# Support for a rare pattern of temperature-dependent sex determination in archaic reptiles: evidence from two species of tuatara (Sphenodon)

**DOI:** 10.1186/1742-9994-3-9

**Published:** 2006-06-29

**Authors:** Nicola J Mitchell, Nicola J Nelson, Alison Cree, Shirley Pledger, Susan N Keall, Charles H Daugherty

**Affiliations:** 1 School of Biological Sciences, Victoria University of Wellington, P.O. Box 600, Wellington, New Zealand; 2 Department of Zoology, University of Otago, Box 56, Dunedin, New Zealand; 3 School of Animal Biology, The University of Western Australia, Crawley 6009, Western Australia, Australia

## Abstract

**Background:**

The sex of many reptiles is determined by the temperature an embryo experiences during its development. Three patterns of temperature-dependent sex determination (TSD) have been defined, but one pattern where only males are produced above an upper temperature threshold (Type IB) is controversial. Here we report new data on the relationship between constant temperature incubation and sexual phenotype in two species of tuatara (*Sphenodon*), archaic reptiles of enormous zoological significance as the sole representatives of a once widespread reptilian order.

**Results:**

In both species, the pattern observed with constant incubation temperatures from 18 to 23°C (or 24°C) supported a female→male (FM or Type IB) pattern of TSD: in *Sphenodon guntheri *males were produced above a pivotal temperature of 21.6°C, and in *S. punctatus *(unnamed subspecies on Stephens Island, Cook Strait), males were produced above a pivotal temperature of 22.0°C. The pivotal temperatures and scaling parameters differed between species (p < 0.001). The thermosensitive period (TSP), where temperature influences gonad morphogenesis, occurs between 0.25 and 0.55 of embryonic development. While it is possible that the more common female→male→female (FMF or Type II) pattern exists, with a second pivotal temperature above 23–24°C, we review several lines of evidence to the contrary. Most notably, we show that in *S. punctatus*, the warmest natural nests during the TSP produce predominantly males.

**Conclusion:**

An FM pattern of TSD could be currently adaptive in promoting sexual size dimorphism in tuatara. However, an FM pattern has particularly serious consequences for *S. guntheri *because current patterns of global warming could exacerbate the male bias already present in the relic population.

## Background

Sex determination in reptiles is remarkably labile, and includes genotypic sex determination (GSD) with or without sex chromosome heteromorphy, parthenogenesis, and temperature-dependent sex determination (TSD) [1]. TSD is a form of environmental sex determination that occurs when embryonic incubation temperatures affect the sex of hatchlings during a critical window of development known as the thermosensitive period (TSP). The TSP coincides with gonad morphogenesis [2], and is generally reported as occurring in the middle-third to middle-half of incubation [3]. Recent *in vitro *and *in vivo *experiments on turtle embryos have shown that bipotential gonads are masculinised in the absence of a temperature trigger during the TSP, whereas female-producing temperatures cause the enzyme aromatase to act locally on gonads to produce oestrogen and activate the development of ovaries [4].

TSD occurs in all four extant orders of reptiles [5-9] including the two species of the archaic order Sphenodontia (tuatara) [9,10]. Tuatara are the only surviving representatives of a once widespread order of lizard-like reptiles that radiated in the Triassic and Jurassic, but that subsequently declined as squamate reptiles became more abundant [11]. The two species are now restricted to island refuges in New Zealand, with 27 populations off the north-eastern coast of the North Island (*Sphenodon p. punctatus*), and five natural populations in Cook Strait (*S. punctatus *unnamed subspecies, and *S. guntheri*) [12]. Genetic diversity among these populations is low for such an ancient order, and mitochondrial DNA data indicate few differences between the geographically proximate *S. guntheri *and *S. punctatus *in Cook Strait [13]. Tuatara eggs have been artificially incubated at constant temperatures since 1985, primarily to provide juveniles for conservation programs. Incubation at 15°C is unsuccessful, and hatching success at 25°C remains equivocal [14], hence intermediate incubation temperatures (18–22°C) with higher hatching success have since been chosen.

Genotypic sex determination does not occur in tuatara; there are no chromosomal differences between females and males [15], nor have any sex-specific DNA markers been identified [16]. Instead, data from the narrow 18–22°C temperature range suggest that tuatara have an extremely rare form of TSD that was first described in squamate reptiles: FM (Type IB) where females result from cool incubation temperatures below about 21°C and males between about 21 and 23°C [9,10]. Two other patterns of TSD are prevalent in other reptile species: MF (Type IA), where males are produced at cooler incubation temperatures, and FMF (Type II), where females are produced at extreme temperatures (within the limits for successful incubation), and males at intermediate temperatures [17]. The MF pattern occurs predominantly in turtles, and the FMF pattern is known from alligators, crocodilians, and from some turtles and lizards (hereon we refer to the three recognised TSD patterns [5] in the intuitive form FM, FMF or MF, rather than Types I and II). The temperatures at which hatchling sex ratios are 1:1 are known as pivotal temperatures, *P *(FM and MF patterns have a single *P*, and FMF patterns have two *P*) and the transitional range of temperatures (TRT) are those at which both sexes can be produced [3].

Previous incubation experiments on the rare North Brother Island tuatara, *S. guntheri*, were ambiguous with respect to the TSD pattern. Constant temperatures of 18° and 22°C produced 100% females, whereas a temperature regime that varied incrementally from 18°→23°→18°C produced 100% males [9]. These data suggested an FM transition above 22°C (if incubation at 23°C coincided with the TSP and so allowed the development of males), or the less likely scenario that the mean of the variable temperature regime (~20°C) triggered males, which would support an FMF pattern. The pattern of TSD in *S. punctatus *is better resolved, but as only one constant temperature has produced exclusively males [10] it remains possible that an FMF pattern could be expressed.

We incubated eggs of *S. guntheri *and *S. punctatus *to compare the pattern of TSD in the two tuatara species within the temperature range 18–23 (or 24)°C. Our new data, combined with data from earlier incubation experiments conducted between 1989–1991 [9] and in 1999 [10] allowed us to evaluate whether an FM or FMF pattern best describes TSD in tuatara, and to estimate the pivotal temperature (or temperatures), and TRT for each species, using a maximum likelihood approach [18,19]. We report *P*, *S *and *K *for each population (see methods for definitions of *S *and *K*) and express TRT as TRT_*x *_– the range of incubation temperatures that produce a sex ratio between *x *and 1-*x*; in most studies *x *is set at 0.05 [18]. Finally, by examining sex ratio and temperature data from natural nests, we provide evidence for FM TSD across a broader range of temperatures.

## Results

### TSD in *Sphenodon guntheri *(North Brother I.)

Sexual phenotype is known for 73 hatchlings incubated at six temperatures, of which two temperatures produced hatchlings of both sexes (Table 1). Minimum theoretical information criteria (AIC) [20] and likelihood ratio test statistics (LRT's) were used to select a TSD model with the best fit; either an FM or FMF model where the inflection around *P *is symmetrical (the logistic models of Girondot [19]) or FM and FMF models where the inflection around *P *is asymmetric (the A-logistic models of Godfrey et al. [18]). For any two models, the model with the lowest AIC fits the data best, but a difference of AIC of less than 3 or 4 signalled that the models were similar in their goodness of fit.

**Table 1:** Incubation temperatures and sex ratios from wild populations of tuatara. t_i _= constant incubation temperature, Ut_i _= uncertainty around t_i _in °C, N_i _= number of sexed embryos, M_i _and F_i _= number of males and females, and Osr_i _= observed male frequency. Numbers of sexed hatchlings from the Little Barrier Island population are included for comparison [10].

*S. guntheri *(North Brother I.)						*S. punctatus *unnamed subsp. (Stephens I.)						*S. p. punctatus *(Little Barrier I.)				
*t*_*i*_	*U t*_*i*_	*N*_*i*_	*M*_*i*_	*F*_*i*_	*Osr*_*i*_	*t*_*i*_	*U t*_*i*_	*N*_*i*_	*M*_*i*_	*F*_*i*_	*Osr*_*i*_	*t*_*i*_	*N*_*i*_	*M*_*i*_	*F*_*i*_	*Osr*_*i*_

18	1	19	0	19	0	18	1	14	0	14	0	18	24	0	24	0
						18.1	0.1	105	0	105	0					
18.3	0.1	12	1	11	0.08											
						20	1	44	3	41	0.07					
20.8	0.1	12	3	9	0.25											
												21	1	0	1	0
						21.3	0.1	80	3	77	0.04					
						21.5*	0.1	7	0	7	0					
22	1	5	0	5	0	22	1	30	9	21	0.3	22	14	3	11	0.21
22.1	0.1	14	14	0	1											
						22.3	0.1	113	113	0	1					
23.0	0.1	11	11	0	1							23	7	7	0	1
						24.1*	0.1	8	8	0	1					

*These eggs were collected from a captive-housed female

**Figure 1:**
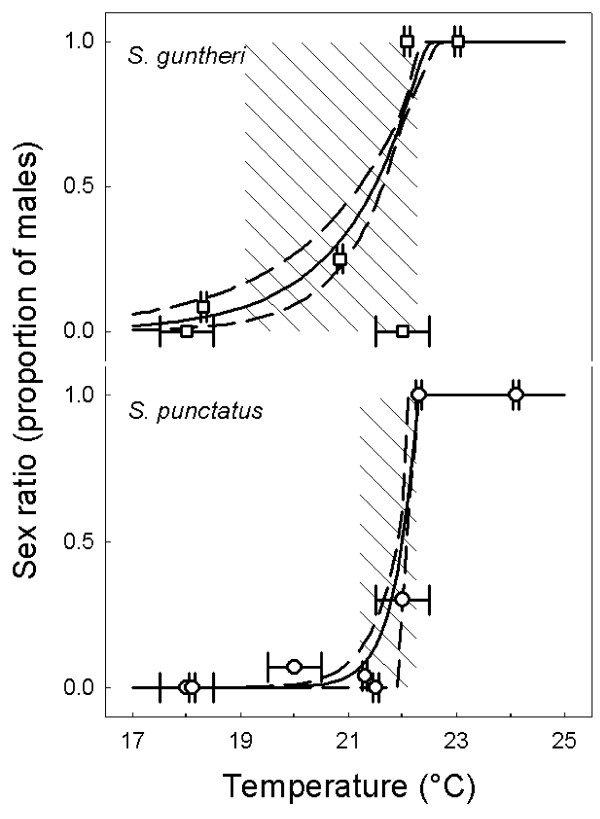
**Relationship between incubation temperature and sex ratio (proportion of males) for two species of tuatara**. Curves (—) are the theoretical function of sex ratio upon incubation temperatures maximizing the likelihood for two experimental data sets: those where incubation temperatures deviated by ± 0.1°C (small error bars) or ± 1°C (larger error bars) from mean temperatures. The TRT_5–95% _is indicated by the hatched lines and dashed lines are ± 2 SE.

In *S. guntheri*, the best model selected by AIC was FM A-Logistic (Figure 1), followed by FM Logistic, FMF Logistic and FMF A-Logistic (with AIC's exceeding the lowest by 1.9, 5.9 and 6.0 respectively). The best model had parameter estimates and their standard errors *P *= 21.57 (± 0.16), *S *= 0.0398 (± 0.1176) and *K *= 3.30 (± 2.94), and TRT_0.05 _= 3.19°C. The FM A-Logistic model was not rejected (p = 0.3561), although the power of the test to detect K ≠ 0 was only 0.4808.

We produced all male hatchlings at 22.1°C, whereas female hatchlings were produced in earlier experiments at 22°C where thermal control was imprecise [9]. The data point of 5 females, 0 males at 22°C (Table 1) was an outlier with a large residual. Its uncertainty of +/- 1°C made it highly influential in AIC model selection. In cases in the uncertainty analysis where the 22°C data point went to its maximum of 23°C and simultaneously the final data point with no females, 11 males at 23°C went to its minimum temperature of 22.9°C, the interchange of the order of the points caused the FMF Logistic model to be selected over the FM Logistic (AIC 24.4 lower on average). Hence, model selection was very sensitive to the magnitude of the uncertainty in incubation temperature. Notably, our estimation of model parameters was reasonably stable if the 22°C outlier was removed: *P *= 21.31, *S *= 0.028 and *K *= 3.60 and TRT_0.05 _= 3.00°C (FM A-Logistic).

### TSD in *Sphenodon punctatus *(unnamed subspecies from Stephens I.)

Sexual phenotype is known for 402 hatchlings incubated at eight temperatures, of which three temperatures have produced hatchlings of both sexes (Table 1). The best model selected by AIC was FM A-Logistic (Figure 1), followed by FM Logistic, FMF A-Logistic and FMF Logistic (with AIC's exceeding the lowest by 6.0, 44.4 and 48.4 respectively). The best model had parameter estimates and their standard errors *P *= 22.03 (± 0.07), *S *= 0.0096 (± 0.0023) and *K *= 3.60 (± 1.94), and TRT_0.05 _= 1.04°C. The FM A-Logistic model was not rejected (p = 0.6354), with power of the test to detect K ≠ 0 being 0.9998. There were no outliers in this data set.

### Comparison of TSD patterns in the two Cook Strait species

A LRT test to compare *S. punctatus *with *S. guntheri *revealed that the fitted parameters differ between the two populations (χ^2 ^= 17.98, 3 d.f., p = 0.0004). All models with P, S and/or K differing or the same were explored, and the best had P and S differing but K the same for the two populations. The next best models had all parameters different (AIC 1.4 higher), or P and K the same but S different (AIC 2.8 higher). This model was also selected by a stepwise procedure with χ^2 ^tests at a 5% significance level. Comparisons of the TSD pattern were not possible between southern tuatara and northern tuatara (*S. p. punctatus*), but for completeness, the sexual phenotype of 46 hatchlings incubated at four temperatures from a population of *S. p. punctatus *from Little Barrier Island [10], are included in Table 1.

### Development times at constant temperatures

**Figure 2:**
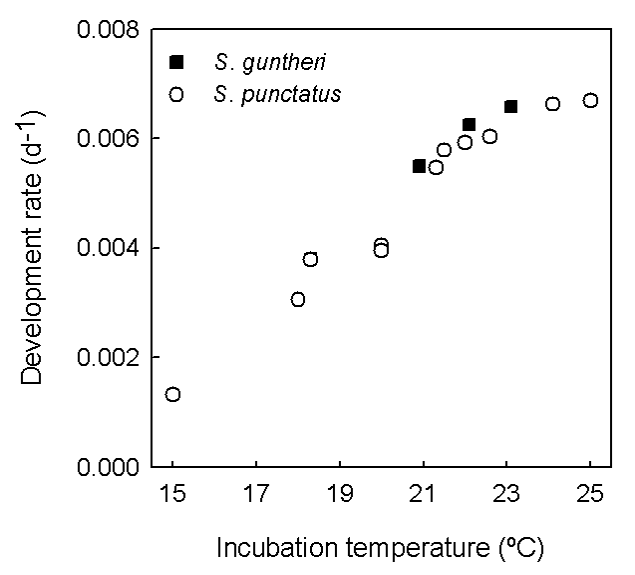
**Relationship between development rate and constant incubation temperature in two Sphenodon species from Cook Strait**. Development rate declines at the hottest incubation treatments attempted for *S. punctatus*, suggesting that the maximum constant temperature tolerated may be only 1–2°C higher.

The time taken to complete development increases with incubation temperature within the entire temperature range examined for each species (Table 2). Development rate (the inverse of development time) increases linearly with temperature between 18–22°C, but slows near 25°C for *S. punctatus *(Figure 2).

**Table 2:** Incubation times at constant temperatures and Q_10 _for development rate for two species of tuatara.

Constant incubation temperature (°C)	Mean days to hatching^a ^(n hatchlings)	Q_10_	Source
*S. guntheri (North Brother I.)*			
18.3	263 (12)		N. Mitchell, this study
20.9	182 (12)	4.14 ^(18.3–20.9)^	N. Mitchell, this study
22.1	160 (15)	2.91 ^(20.9–22.1)^	N. Mitchell, this study
23.1	152 (11)	1.68 ^(22.1–23.1)^	N. Mitchell, this study
			
*S. punctatus (Stephens I.)*			
15	764 (1)^b^		Thompson 1990 [14]
18	328 (29)		Thompson 1990 [14]
18.3	264 (105)	25.03 ^(15–18.3)^	Nelson et al. 2004 [32]
20	247 (12)		Thompson 1989 [44]
20	253 (64)		Thompson 1990 [14]
21.3	183 (80)	3.39 ^(18.3–21.3)^	Nelson et al. 2004 [32]
21.5	173 (5)^c^		A. Cree, this study
22	169 (32)		Thompson 1990 [14]
22.6	166 (113)	2.12 ^(21.3–22.6)^	Nelson et al. 2004 [32]
24.1	151 (5)^c^		A. Cree, this study
25	150 (2)	1.53 ^(22.6–25)^	Thompson 1990 [14]

^a^Days to hatching includes short periods spent in natural nests, in transit from field sites to incubators, or in an 18°C incubator (range 2–20 days)

^b^Incubation time derived from an embryo that pipped the eggshell but did not hatch [14]. The true 15–18.3°C Q_10 _could be lower if this embryo had completed development earlier but could not hatch.

^c^Data from a successful, but as yet unsexed clutch

### Thermosensitive period

**Figure 3:**
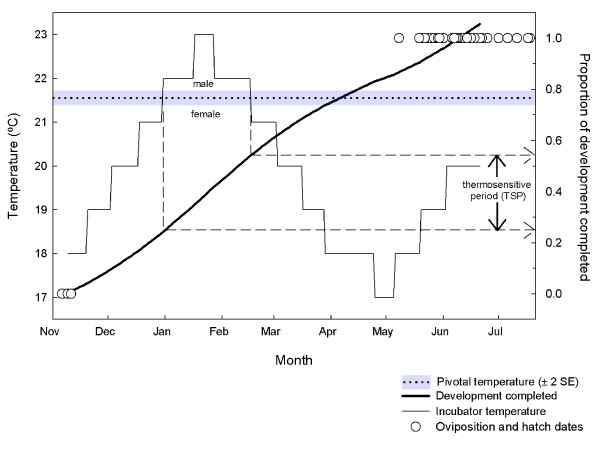
**Development of *S. guntheri *embryos under a temperature regime designed to mimic the natural incubation environment**. These experiments, conducted in three successive years (1989–1991), always produced males. The proportion of development completed (heavy line) was estimated from development rates at constant temperatures adjusted to set incubator temperatures using Q_10 _for *S. guntheri*, and a Q_10 _for *S. punctatus *for temperatures below 18°C (Table 2). Because incubator temperatures only exceeded the pivotal temperature (21.57°C) over a single developmental window, we infer that the TSP for tuatara occurs wholly or partially within 0.25 and 0.55 of development.

Our estimation of the pivotal temperature for sex determination in *S. guntheri *allows us to estimate the TSP with reference to early incubation experiments with *S. guntheri *embryos where temperatures were increased and then decreased to mimic natural incubation temperatures. These experiments (conducted between 1989 and 1991 [9]) produced 100% male hatchlings, and temperatures exceeded the pivotal temperature of 21.57°C for almost seven weeks. It is now evident that this period spent at male-producing temperatures fell within the TSP (Figure 3).

In order to identify when in development tuatara embryos are sensitive to temperature we first calculated rates of development at constant temperatures as the inverse of known development times (Table 2). Then, for each day of incubation, we calculated the proportion of development completed, which was the inverse of the development rate calculated for the incubator temperature on that day, adjusted using the Q_10 _in Table 2. We then added the development completed each day to the previous days' tally, and produced a curve that related the proportion of development completed with temperature, where 1 denoted hatching (Figure 3). We found a good match between the predicted hatch dates and the actual dates that embryos hatched. This gave us confidence to infer that 25% of embryonic development had been completed by the beginning of the seven-week thermosensitive period, while 55% of development was completed at the end of the period. Hence the TSP falls within 0.25 and 0.55 of embryonic development in tuatara, although the TSP may not be entirely restricted to this window.

### Evaluation of the TSD pattern using data from natural nests

Natural incubation temperatures and sex ratios are known for 23 nests of *S. punctatus *on Stephens Island [10]. Briefly, small temperature loggers (Hobo Stowaway: Onset) were inserted amongst eggs (distributed in 1–3 layers) and recorded temperatures each hour for the duration of development. Three nests failed completely, and in four nests only one egg hatched. Of these seven nests, two had the coolest mean temperature, and two had the highest mean temperature. However, high hatching success (80 and 100 %) occurred in two nests that had the highest daily maxima (34.4 and 34.1°C), suggesting that short-term exposure to extreme temperatures does not kill tuatara embryos. These two nests produced 100% and 88% males respectively.

Exposure of turtle embryos to female-producing temperatures for as little as four days during the TSP is sufficient to irreversibly determine sex [4], hence we elected to explore the relationship between nest temperatures and the sex ratio response over small development windows within the putative thermosensitive period, ranging from 5 to 30% of embryonic development (Table 3). We calculated a 'constant temperature equivalent', or CTE, for each day of development [21]. Then, for each nest we identified the dates that encompassed a particular developmental window (e.g. 0.50–0.55) after plotting the cumulative development completed since oviposition, (calculated using development rates and Q_10 _in Table 2 – see earlier) against the daily CTE. We then averaged daily CTE's over each period to produce a temperature covariate for model fitting.

**Table 3:** Relative AIC values over 21 development windows (0.25–0.30, etc.) and four model types.

Embryonic development window	Proportion of embryonic development period	TSD Model				Mean
			
		FM.L	FM.A	FMF.L	FMF.A	
0.25 – 0.30	5%	15	16.5	17.6	15.5	16.2
0.30 – 0.35		28.2	30.2	32.2	36.2	31.7
0.35 – 0.40		4.3	6.3	1.6	4.1	4.1
0.40 – 0.45		35	36.8	35.7	38.3	36.5
0.45 – 0.50		20.3	22.1	22.7	27	23.0
0.50 – 0.55		0	1	4	7	3.0
	
0.25 – 0.35	10%	14.9	16.6	17.2	14.1	15.7
0.30 – 0.40		14.5	16.5	18.5	22.5	18.0
0.35 – 0.45		17.8	19.5	15	17.3	17.4
0.40 – 0.50		21.9	23.8	23.1	20.2	22.3
0.45 – 0.55		10.5	12.4	13.6	15.5	13.0
	
0.25 – 0.40	15%	7.5	9.4	7.7	7.2	8.0
0.30 – 0.45		16.3	18.2	15.2	15.8	16.4
0.35 – 0.50		14.9	16.8	13.8	16.9	15.6
0.40 – 0.55		12.6	14.4	14.3	16.1	14.4
	
0.25 – 0.45	20%	10.4	12.2	9.6	8	10.1
0.30 – 0.50		14	15.9	14.1	15.4	14.9
0.35 – 0.55		9.3	11.2	8.3	10.7	9.9
	
0.25 – 0.50	25%	11.4	13.2	11.9	12.6	12.3
0.30 – 0.55		9.6	11.5	10.6	13.2	11.2
	
0.25 – 0.55	30%	9	10.8	9.9	11.1	10.2
	
	Mean	14.2	16.0	15.1	16.4	

FMF.L = FMF logistic, FMF.A = FMF A-logistic, FM.L = FM logistic and FM.A = FM A-logistic.

For the sex ratio response data from the 16 nests that produced more than one hatchling, we fitted 84 maximum likelihood models, comprising one of four TSD models (FMF logistic, FMF A-logistic, FM logistic or FM A-logistic), and one of 21 temperature covariates calculated from the different development windows, as described above. The 84 different models were compared by relative AIC, which is AIC – minimum (AIC).

**Figure 4:**
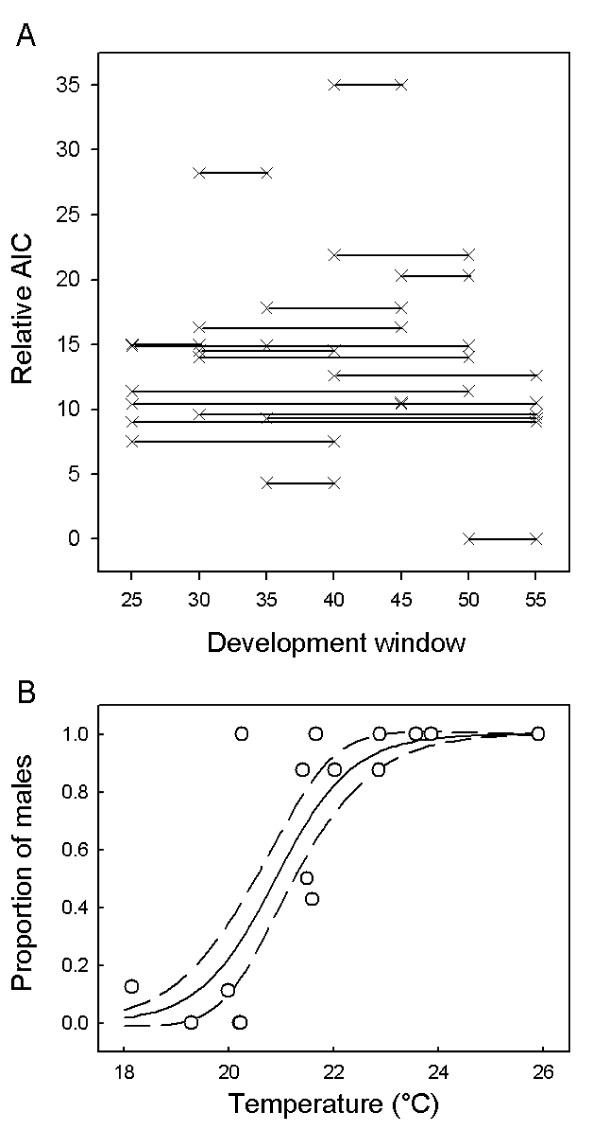
**A. Relative AIC over different development windows, using TSD model type FM logistic**. The period 0.50–0.55 has the lowest AIC (relative AIC set to zero). Generally development windows including 0.50–0.55 have low AIC. **B. Example showing the relationship between the CTE (0.50–0.55) in natural nests and the hatchling sex ratio**. The curve (—) is the maximum likelihood fit, and dashed lines are ± 2 SE.

The best fitting model was that which used the CTE over 50–55% of development and model FM logistic (Table 3, Figure 4), where the estimated parameters were (± 1 standard error) of pivotal temperature *P *= 20.89 (± 0.18) and *S *= 0.72 (± 0.13). However, other models (50–55 FM.A and 35–40 FMF.L) showed similar goodness of fit. Totalling over the rows supported the 0.50–0.55 development window having the best relationship to hatchling sex over a range of model types (lowest row total), and the lowest column total indicates that model type FM logistic had the highest support over a range of possible development windows (Table 3).

Figure 4 shows the relative AIC values for the different development windows, using the FM logistic model. Although the 0.50–0.55 development window exhibits the lowest AIC, there is no clear picture confirming it as the single most important period. Other short periods showing low AIC included 35–40%, but AIC were high over other short periods (notably 0.30–0.35, 0.40–0.45 and 0.45–0.50).

## Discussion

### Interpopulation differences in TSD patterns

The two study populations in Cook Strait have similar pivotal temperatures (*P*): 21.57°C for *S. guntheri *on North Brother Island, and 22.00°C for *S. punctatus *on Stephens Island. Data are too few for northern tuatara, but *P *appears to fall between 22 and 23°C (Table 1). A general pattern in other populations of reptiles with TSD is that substantial intraspecific variation in pivotal temperatures exists in large populations with high genetic diversity [19,22-24] and should result in a larger TRT relative to small populations. Our estimate of the TRT for Stephens Island tuatara, which represents the largest extant population of around 30 000 individuals [12], was 1.1°C. Hence, because the narrow TRT occurred in the largest tuatara population, it is likely to be characteristic of tuatara, rather than a small population effect. Conversely, the estimated TRT of 3.2°C in *S. guntheri *was partly due to the contrary results from incubation at 22°C, and future incubation experiments closer to the pivotal temperature will probably also reveal a TRT of around 1°C. Notably, a TRT of 1°C is amongst the narrowest known for any reptile population [19,25] and may leave tuatara particularly vulnerable to the demographic effects of global warming, as larger TRT should endow greater resilience to changing environmental temperatures [24].

### Evidence for FMF TSD in tuatara?

The FM pattern is exceptionally rare among reptiles. Given that most reptiles originally thought to have MF or FM sex determination have proven to have FMF TSD when a wider range of incubation temperatures was examined [18,26], it is worth considering this possibility for *Sphenodon*. A study of leopard geckos demonstrated that if temperatures were dropped by as little as 2°C prior to hatching, embryos could survive incubation at temperatures that were previously thought to be too high for production of viable hatchlings [26]. Notably, these experiments were responsible for the revision of the TSD pattern from FM to FMF, and similar experiments should be attempted for tuatara to assess the possibility of an FMF pattern.

A key question is what is the upper temperature limit for successful incubation in tuatara? An early study suggested that the upper lethal limit for constant temperature incubation in Cook Strait *S. punctatus *is close to 25°C [14], but imprecise temperature control in the incubators and uncertainties about egg quality mean that the true tolerance limit may be higher. An indication of the upper temperature limit for the successful incubation of tuatara embryos may be a decline in development rate [27]. Available data show that development rates at the highest constant incubation temperatures attempted for *S. punctatus *are lower than would be expected from a linear relationship (Table 2, Figure 2), suggesting that the upper temperature limit for successful incubation is close to being reached at 25°C. However, under natural conditions, tuatara hatch successfully from nests that experience repeated exposure to temperatures above 30°C [10,28].

Sex ratio data from the hottest field nests provided an excellent opportunity to assess the likelihood of FMF TSD in tuatara, just as high cycling temperatures served to demonstrate FMF TSD in the agamid *Amphibolurus muricatus *[29]. Our exploratory analyses supported an FM pattern over an FMF pattern, but there was too much sampling fluctuation for a precise TSP to be identified. Determining the exact boundary of the TSP will require switch-switch experiments [8], and our analysis of field data should be a useful guide when selecting an appropriate period of development to manipulate in the laboratory. Further, precise knowledge of the TSP will be critical to accurately predicting sex ratios arising from natural nests.

Survival of reptile embryos is a product of nest temperature and moisture content, and desiccation is a major cause of egg failure in natural nests of tuatara [28]. Because of the high survival rates in the hottest tuatara nests, it is unlikely that our support for an FM pattern is a consequence of temperature-dependent mortality. An FMF pattern could still be expressed in tuatara if even hotter nests produced predominantly female hatchlings, but extremely hot nests would occur only in shallow exposed soils where eggs are prone to desiccation-induced mortality. If moisture could be kept sufficiently high, it may be possible for female hatchlings to be produced at even higher temperatures. However, unlike cases with crocodilians that have FMF patterns of TSD [30], hot, moist tuatara nests have not been found to occur in nature [28].

### Evidence for FM TSD in other reptiles

Experiments on the thermal effects of egg incubation are most complete for chelonians (12 of 13 families, or 30% of turtle species), where the sex determination pattern is predominantly MF or FMF, or occasionally GSD [25]. An FM pattern was first reported in some squamates and crocodilians, but in well-studied species, subsequent experiments spanning a broader range of incubation temperatures have without exception revealed FMF TSD (Table 4). FMF TSD is presumed to occur in all Crocodilians [30], but as limited data available for *Alligator sinensis, Caimen crocodilus yacare *and *Paleosuchus trigonatus *still suggest FM TSD, further experiments are required to confirm the uniformity of the FMF pattern in Crocodilians.

**Table 4:** Studies of reptiles reporting FM TSD where the pattern was later revised to FMF TSD.

Species	Studies reporting FM	Studies reporting FMF	Consensus
Squamata			
*Agama agama*	Charnier 1966 [45]	El Mouden et al. 2000 [46]*	FMF
*Ctenophorus decresii*	Godfrey et al. 2003 [18]	Harlow 2000, 2004 [31, 47]	FMF
*Eublepharis macularius*	Wagner 1980, Bull 1987a, 1987b [2, 48, 49]	Viets et al. 1993 [26]	FMF
			
Crocodilians			
*Alligator mississippiensis*	Deeming and Ferguson 1989, 1991 [50, 51]	Lang and Andrews 1994 [6]	FMF
*Caiman crocodilus crocodilus*	Lang et al. 1989 [52]	Lang and Andrews 1994 [6]	FMF

*FMF pattern inferred for *A. agama *based on data for *A. impalearis*

Among the squamates, all orders appear to have either GSD or FMF TSD [31]. The pattern of TSD in Australian agamid lizards is well defined for some species, but insufficiently studied in others where available data suggests FM TSD [31]. In this group the FMF pattern of TSD is characterised by a broad TRT and an upper temperature band only 1–2°C wide where 100% females are produced, and above which all embryos die. In many cases incubation of embryos at temperatures fluctuating about the upper limit for constant temperature development have been critical to revealing an FMF pattern [29,31]. The existence of FMF TSD and GSD in closely related agamids (e.g. *Amphibolurus muricatus, A. nobbi *and *A. norrisi*), suggests that the pattern of sex determination has current or past adaptive value in this group [31].

### Could FM TSD be adaptive in tuatara?

Incubation duration does not affect the size of hatchling tuatara, but incubation temperature may have an indirect effect on the size of juveniles by controlling variation in hatching time in nature [32]. Hatching occurs over at least five months after a total incubation duration time of at least 11 months [33] (NJN, pers. obs.). Male tuatara hatch earlier, and may have a greater amount of time before winter to find refuges and grow. Sex-specific advantages resulting from spatial or temporal patches in the environment are proposed to support not only the origin, but the maintenance of TSD through their longer term influence on fitness [34]. For example, adult male tuatara are larger than females, and larger males may gain greater access to mates, providing a plausible explanation for the adaptive advantage of an FM pattern of TSD [8,34,35]. However, these effects can be removed when juveniles are reared in similar conditions in captivity: females produced from constant incubation at 21°C are larger by ten months of age than males from 22°C [32]. The current adaptive advantage of an FM pattern of TSD is questionable in the face of rapid temperature increases predicted as a result of global warming [36]. A male-biased adult population of *S. guntheri *already exists on North Brother Island [37], and although nest site choice by female tuatara is the subject of further research, early indications are that their nesting behaviour will not correct this imbalance [38].

## Conclusion

The sexual phenotypes resulting from constant-temperature incubation in two tuatara species were significantly better described by an FM pattern than an FMF pattern, and the response to temperature was significantly different between species. Further experiments are needed to unequivocally rule out an MF transition (second pivotal) above 24°C, but field data for *S. punctatus *suggest that a second transition is unlikely. An FM pattern of TSD is particularly significant because male-biased sex ratios are already evident in natural populations of tuatara [37], and global warming may further skew sex ratios towards males.

## Methods

### Egg incubation at constant temperatures and sexing of hatchlings

Details of egg incubation and hatchling husbandry for Stephens Island *S. punctatus *are reported elsewhere [32]. Here we describe new incubation experiments using wild-collected eggs of the Brother's Island tuatara, *S. guntheri*, and eggs from a captive female *S. punctatus *that originated from Stephens Island.

#### *Sphenodon guntheri *(North Brother Island)

The only natural population of *S. guntheri *occurs on North Brother Island, Cook Strait, New Zealand. We collected 71 eggs of *S. guntheri *from North Brother Island in December 2000 by inducing oviposition in gravid females with an intraperitoneal injection of oxytocin (Oxytocin-s, conc. 10 IU/mL, Intervet International BV, Boxweer, Holland; [39]), or by removing eggs from freshly completed nests. Twenty-two eggs were removed from four natural nests in December 2000 (eggs oviposited between 22 November and 5 December 2000), while 49 eggs were obtained from inductions on 7–10 December 2000. Nine of 21 females induced with oxytocin began to deposit clutches of 3–7 eggs within 15 to 70 min of the injection. Two females produced small soft eggs that were not completely calcified and that did not develop successfully. The remaining females exhibited no response to oxytocin, despite the apparent detection of palpable eggs.

All eggs were assigned a unique number on their upper surface with a soft graphite pencil, and immediately transferred to moist vermiculite (-170 kPa water potential, or 80% water by dry mass of vermiculite) in 2 L plastic containers. Eggs were transported to constant temperature incubators at Victoria University of Wellington within 2–20 d of oviposition (median 3 d), where they were stratified by clutch, and randomly assigned to a constant temperature incubator set to 18°, 21°, 22° or 23°C. Incubators were calibrated prior to use, and incubation temperatures were monitored daily with maximum/minimum thermometers. Incubation temperatures were also recorded by dataloggers placed among eggs (waterproof Stowaway^® ^Tidbit^® ^temperature recorders, Onset Computer Corporation, Massachusetts, USA; hourly recordings). Incubation boxes were moved within each incubation chamber each week to offset any effects of thermal stratification within the incubators.

Viable eggs were weighed weekly, whereas eggs that ceased to develop (lost mass or became mouldy) were removed. Distilled water was added to incubation boxes to compensate for small losses from the container and uptake by the eggs, thus maintaining a water potential of approximately -170 kPa. At hatching, tuatara were marked with a unique toe-clip, and transferred to 600 × 700 mm enclosures in groups of 15–16 individuals, grouped according to hatch date [32].

Juvenile tuatara cannot be sexed externally with confidence until they attain approximately 130–160 mm SVL (AC, unpublished observation), so hatchlings were sexed when aged approximately one year by internal examination of the right gonad using a laparoscope [10,40]. The procedure involved swabbing the right lateral surface of the abdomen with 70% ethanol and injecting local anaesthetic (2% lignocaine hydrochloride; approx. 0.05 ml per animal) into abdominal musculature, or as a small bleb under the skin if juveniles were less than 10 g body mass. A 2 mm incision was made through the body wall at the site of injection, approximately one-third of trunk length anterior from the rear legs. Reproductive organs (gonads, and reproductive ducts where visible) were examined using a 1.7 mm diameter endoscope with 10× magnification (Olympus A7002, Japan), and gonad sex ascertained. The incision was sutured with absorbable chromic cat gut, and the area dusted with antibiotic powder. Each operation took approximately 5 minutes. All equipment and consumables that were not already sterile were soaked in a non-selective, biodegradable antiseptic.

#### *Sphenodon punctatus *(unnamed subspecies from Stephens Island)

Fifteen eggs laid in captivity by one female *S. punctatus *at the Southland Museum and Art Gallery on 1 October 2003 were held at 18°C for seven weeks before transportation to the University of Otago, where they were incubated at 21.5°C (n = 7) or 24.1°C (n = 8) in moist vermiculite. Based on the mean hatch time for *S. punctatus *of 328 days at 18°C (Table 2), these embryos would have progressed 0.15 toward hatching (49 days of development at 0.003 d^-1^) and gonads should still have been bipotential when they were switched to the experimental temperature. The experimental temperatures were chosen to approximate the pivotal temperature (21.5°C), and to determine whether a warmer constant incubation temperature than previously known to be viable for *S. punctatus *(24.1°C) would produce males. The external probe of a temperature logger (Stowaway^®^) was placed into the vermiculite in each box and temperature was recorded every 15 minutes.

All eggs in this clutch failed at a very late stage of incubation (late Stage R-Stage S on the scale of Dendy [41], equivalent to about early Stage 40 on the Dufaure and Hubert scheme for *Lacerta vivipara *[42]). All but one of the eggs developed splits in the shell between 20 February and 10 April 2004, and the remaining egg was cut open on 26 March 2004. The embryos had spinal deformities and large yolk sacs, and were either dead or incapable of sufficient movement to pip and emerge from the shell. The failure of these embryos is suspected to involve a calcium deficiency, and was not temperature-related because another clutch incubated at the same temperatures was 100% successful (the resulting hatchlings are, as yet, too small to sex). Embryos from the failed clutch were euthanized where necessary with halothane vapour, and preserved in neutral buffered formalin. The posterior abdomen was later embedded in paraffin, serially cross-sectioned and stained using H&E, allowing gonad sex to be determined.

### Model selection and determination of pivotal temperatures and TRT

We used the program TSD version 3.5.1  for our analyses; it requires that mixed sexes be produced at two or more incubation temperatures, and enables the user to define uncertainty about the true mean incubation temperature [18]. In the earlier (1989–1991) studies thermal control was imprecise; hence we assumed an uncertainty of ± 1.0°C. In contrast, we assumed an uncertainty of ± 0.1°C in experiments conducted since 1999, based on incubation temperatures measured each hour, or more frequently. Further, in all cases, we calculated the mean temperature for the middle half of incubation (rather than the mean temperature of the entire incubation period), as gonad differentiation occurs mid-incubation in other reptiles with TSD [43].

We fitted four TSD models to data for each species: FM and FMF logistic models [19], and FM and FMF modified A-logistic models [18]. In brief, logistic models describe sex ratio, *sr *(expressed as the proportion of male hatchlings) as a function of constant incubation temperature (t) as follows:

sr (*t*) = (1 + *e*^(1/*S*•(*P*-*t*))^)^-1 ^    (1)

where *S *defines the shape of transition from one sex to the other, and *P *is the pivotal temperature. S is >0 for FM TSD and <0 for MF TSD, and is symmetrical around *P*. To account for the possibility of asymmetry around *P*, the A-logistic equation introduces the parameter *K *to define the asymmetrical shape [18]:



When *K *= 0 then equation (2) reduces to equation (1). We implemented the option to estimate the power of the test to detect K ≠ 0, which allowed us to determine whether the selection of symmetry about *P *was an artefact of insufficient data [refer [18]]. The relationship between the TRT and *S *is defined as TRT_l _= |*S*·K_l_|, where K_l _is a constant equal to [2·*ln *(l/(1 - l))] [19]

## Competing interests

The authors declare that they have no competing interests.

## Authors' contributions

NJM carried out the work on *S. guntheri *and drafted the manuscript; NJN conducted the laparoscopies, and helped to draft the manuscript. AC conducted experiments on *S. punctatus *that provided additional support for the FM TSD pattern. SP performed the statistical analysis, SNK coordinated fieldwork and assisted with husbandry of juvenile tuatara, and CHD conceived the study at Victoria University of Wellington. All authors read, commented on, and approved the final manuscript.
